# NGS-Based Mutation Profiling and PD-L1 Expression in NSCLC Patients: A Single-Centre Prospective Analysis

**DOI:** 10.5146/tjpath.2026.14900

**Published:** 2026-09-15

**Authors:** Anab Sayyada, Dheeraj Gautam, Rashi Sharma, Apeksha Bhat, Jyoti Wadhwa, Mamta Arya

**Affiliations:** Department of Pathology and Lab Medicine, Medanta-The Medicity, Haryana, İndia; Department of Medical Oncology & Haematology, Paras Health, Haryana, İndia; Molecular Lab (Pathology), Rajiv Gandhi Cancer Institute & Research Centre, New Delhi, İndia

**Keywords:** Non-small cell lung cancer (NSCLC), Next-generation sequencing (NGS), Actionable mutations, PD-L1 Expression, EGFR, KRAS mutations

## Abstract

Objective: Non-small cell lung cancer (NSCLC) is a molecularly heterogeneous disease in which both actionable mutations and PD-L1 expression influence therapeutic decisions. This study aimed to evaluate the molecular profile of NSCLC using next-generation sequencing (NGS) and analyse the association of PD-L1 expression with key genetic alterations.

Material and Methods: A retrospective analysis was conducted on 87 histopathologically confirmed NSCLC cases. Molecular profiling was performed using the Oncomine™ Lung Focus Assay, which targets major actionable mutations. PD-L1 expression was assessed by immunohistochemistry (IHC) using the Tumour Proportion Score (TPS) and categorised as <1% (negative), 1- 49% (weak positive), and ≥50% (strong positive).

Results: A total of 105 molecular alterations were identified across 87 cases, with EGFR being the most frequently mutated gene (36.2%), followed by KRAS (16.2%) and AR amplification (14.3%). Actionable mutations were defined as alterations with approved targeted therapies or clinical trial eligibility were detected in 59.8% of patients, with EGFR exon 19-21 being the most frequent (25.7%), followed by ALK fusions (5.7%), ERBB2 exon 20 (4.8%), KRAS G12C (3.5%), MET exon 14 skipping (2.9%), and BRAF V600E and ROS1 (1.9% each). PD-L1 expression was observed in 45.7% of cases. PD-L1 positivity was lower in EGFR-mutant tumours compared to EGFR wild-type, suggesting reduced immunogenicity in this subgroup. Conversely, KRAS-mutant tumours exhibited higher PD-L1 expression than KRAS wild-type tumours, suggesting a potential predictive role for immunotherapy. ALK-rearranged tumours showed variable but notable PD-L1 expression.

Conclusion: The study underscores the importance of integrating NGS-based molecular testing with PD-L1 evaluation for personalised management of NSCLC. Distinct patterns of PD-L1 expression across molecular subtypes, particularly lower in EGFR-mutated tumours and higher in KRAS-mutated tumours, underscore the need for tailored therapeutic strategies and informed sequencing of targeted therapies and immunotherapies.

## Introduction

In 2020, lung cancer was the most diagnosed cancer globally, with 2.5 million new cases (12.4% of all cancers) and the leading cause of cancer deaths, with 1.8 million fatalities (18.7%) (1). Cigarette smoking is the primary cause, linked to 80% of cases, with a clear dose-response relationship (2). Other risk factors include air pollution, second-hand smoke, and occupational exposures (asbestos, uranium) (3).

Lung cancer is divided into non-small-cell lung cancer (NSCLC, 85%) and small-cell lung cancer (SCLC, 15%) (4). NSCLC encompasses adenocarcinoma, squamous cell carcinoma (SCC), large cell carcinoma (LCC), and other rare subtypes (5). Adenocarcinoma is the predominant histologic type, often occurring in women, non-smokers, and younger individuals, typically originating in the lung periphery (2). SCC is strongly associated with tobacco exposure and involves the central airways (2). Adenocarcinoma shows immunohistochemical (IHC) expression of markers such as thyroid transcription factor 1 (TTF1) and napsin A, whereas SCC exhibits expression of markers including p40, p63, and cytokeratin 5/6. LCC and other uncommon variants are generally diagnosed by exclusion in the absence of specific differentiation markers (5). SCLC is aggressive, nearly exclusive to smokers with neuroendocrine features.

At the molecular level, lung carcinogenesis arises through the accumulation of oncogenic driver mutations. Adenocarcinomas frequently have alterations in receptor tyrosine kinase signalling genes such as EGFR (15% in Western populations and up to 62% in Asian populations), ALK (3–5%), ROS1 (1%), MET (2-5%), and RET (1-2%) (2,6). Additional mutations include BRAF (2%), PI3K (2%), and KRAS (20–25%) (7). SCC is characterised by molecular mutations involving CDKN2A, TP53 mutations, and FGFR amplification. SCLC carries the highest mutational burden, typically exhibiting inactivation of TP53 and RB1 (2,7). Globally, around 25% of lung cancers occur in never-smokers and predominantly affect women and are most often adenocarcinomas harbouring targetable mutations such as EGFR, whereas KRAS alterations are rare (2).

All patients with advanced NSCLC should undergo molecular testing to identify actionable mutations. Current guidelines from the College of American Pathologists (CAP), International Association for the Study of Lung Cancer (IASLC), and the Association for Molecular Pathology (AMP) recommend testing either the primary tumour or a metastatic site and testing should include EGFR, ALK, ROS1, BRAF, KRAS, MET, RET, and ERBB2 (HER2), preferably via the NGS panel in all NSCLC cases with any adenocarcinoma component or in patients with a light or never-smoking history (8,9).

Techniques for molecular diagnostics include direct Sequencing, PCR testing, NGS, Fluorescence In Situ Hybridisation (FISH), and IHC. DNA and RNA-based NGS enable the detection of both known and novel genetic alterations. RNA-NGS is particularly valuable for identifying gene fusions, such as MET exon 14 skipping. Additionally, circulating tumour DNA (ctDNA) in plasma can be analysed using NGS to detect molecular rearrangements (7,10). IHC is an accepted screening alternative to FISH for ALK detection, offering rapid results within 24 hours. IHC is the approved method for PD-L1 assessment (7,11). PD-L1 (CD274/B7–H1) is a transmembrane protein that mediates immune suppression by binding to PD-1 and inhibiting effector T-cell activity, allowing tumour immune evasion. It is expressed on immune and tumour cells and is frequently upregulated in NSCLC. Blockade of the PD-1/PD-L1 pathway restores antitumour immunity, forming the basis for immune checkpoint therapy (12). Clinical trials have shown that high PD-L1 expression in advanced NSCLC correlates with improved outcomes to pembrolizumab compared with chemotherapy. Clinically, PD-L1 serves as a key biomarker for selecting patients for immunotherapy (13).

Molecular insights have revolutionised NSCLC management by enabling targeted therapies such as monoclonal antibodies and tyrosine kinase inhibitors (TKIs), which selectively inhibit oncogenic drivers like EGFR and ALK. For cases resistant to chemotherapy or exhibiting immune evasion, immune checkpoint inhibitors offer significant benefit (14).

## Material and methods

### Study Design and Population

A retrospective observational study was conducted at a tertiary care centre between January 2023 and March 2024. A total of 87 consecutive adult patients with histologically confirmed NSCLC were enrolled. Histopathological diagnosis was established based on tumour morphology and confirmed with relevant IHC markers as per standard practice. Molecular profiling by NGS and PD-L1 IHC assessment were performed as part of the routine diagnostic workup at the time of initial pathological evaluation. Cases were excluded if the patient was under 18 years of age, had a diagnosis of small-cell lung cancer, or had an NSCLC diagnosis without an available NGS report.

### Sample Types and Tissue Processing

All eligible patients underwent biopsy from the appropriate site (primary tumour, regional lymph node, or metastatic site) for pathological assessment and tissue diagnosis. NGS testing was performed on formalin-fixed paraffin-embedded (FFPE) tissue obtained from 87 cases, comprising 72 tru-cut biopsies (82.8%), 12 surgical resection specimens (13.8%), and 3 cell blocks (3.4%). Representative tumour-rich areas were identified and marked by the reporting pathologist, and 3-5 FFPE tissue and cell block sections were used for nucleic acid extraction, requiring as little as 10 ng of input DNA or RNA per reaction.

### Next-Generation Sequencing (NGS)

NGS was performed on all eligible NSCLC samples to detect actionable molecular alterations using the Oncomine Focus Assay (Thermo Fisher Scientific). This assay is based on Ion AmpliSeq technology and enables highly multiplexed analysis of both DNA and RNA in a single workflow to detect hotspot single-nucleotide variants (SNVs), insertions/deletions (indels), copy number variations (CNVs), and gene fusions across a targeted 52-gene panel (Table 1).

**Table 1:** Oncomine Focus Assay: 52-Gene Panel

**Hotspot Genes (SNVs/Indels)**	**Copy Number Variants**	**Fusion Drivers**
AKT1, ALK, AR, BRAF, CDK4, CTNNB1, DDR2, EGFR, ERBB2, ERBB3, ERBB4, ESR1, FGFR2, FGFR3, GNA11, GNAQ, HRAS, IDH1, IDH2, JAK1, JAK2, JAK3, KIT, KRAS, MAP2K1, MAP2K2, MET, MTOR, NRAS, PDGFRA, PIK3CA, RAF1, RET, ROS1, SMO	ALK, AR, BRAF, CCND1, CDK4, CDK6, EGFR, ERBB2, FGFR1, FGFR2, FGFR3, FGFR4, KIT, RAS, MET, MYC, MYCN, PDGFRA, PIK3CA	ABL1, AKT3, ALK, AXL, BRAF, EGFR, ERBB2, ERG, ETV1, ETV4, ETV5, FGFR1, FGFR2, FGFR3, MET, NTRK1, NTRK2, NTRK3, PDGFRA, PPARG, RAF1, RET, ROS1

The NGS workflow comprised four sequential steps. First, DNA and RNA were extracted from FFPE tissue sections containing adequate tumour content. Library preparation was then performed using the Oncomine Focus Assay kit, which involved target amplification using 52 primer pairs in a single pool, adapter and barcode ligation, purification of the unamplified library, library amplification, and purification of the amplified library. The amplicon length ranged from 111 to 187 bp (average 154 bp). Second, template preparation was carried out using the Ion Chef Instrument with the Ion One Touch OT2 system (emulsion PCR) and the Ion One Touch enrichment kit. Libraries were pooled, loaded into the emulsion PCR system, and following enrichment to select template-positive ion sphere particles, the enriched library was stored at 2–8°C until sequencing. Third, sequencing was performed on the Ion Gene Studio S5 semiconductor sequencing platform (Thermo Fisher Scientific) using the Ion 520 Chip, accommodating 6 samples and 2 controls per run. The average depth of coverage achieved was >2000×, with >96% on-target reads and a 98% SNP detection sensitivity. The turnaround time from sample to results was approximately 3 days. Fourth, post-sequencing variant identification and prioritisation were performed using Ion Reporter Software, and the Ion Torrent Oncomine Reporter was utilised to generate comprehensive reports linking identified biomarkers to relevant clinical evidence and therapeutic implications.

### PD-L1 Immunohistochemistry

PD-L1 expression was assessed using the PD-L1 IHC 22C3 pharmDx assay (SK006, Dako/Agilent), a qualitative immunohistochemical assay employing anti-PD-L1 antibody, Clone 22C3, on FFPE tumour tissue and cell block sections using the Autostainer Link 48 platform. Only sections containing a minimum of 100 viable tumour cells were considered adequate for evaluation; necrotic areas were excluded from scoring. PD-L1 expression was evaluated by the reporting pathologist using the tumour proportion score (TPS), defined as the percentage of viable tumour cells showing partial or complete membranous staining at any intensity (≥1+) relative to all viable tumour cells in the sample. Only membranous staining of tumour cells was scored; cytoplasmic staining was excluded. Benign human tonsil tissue served as the recommended positive control. PD-L1 status was classified into three categories: negative (TPS <1%), weak positive (TPS 1- 49%), and strong positive (TPS ≥50%) (15). PD-L1 expression data were unavailable for 17 cases (19.5%).

### Statistical Analysis

Descriptive statistics were used to summarise clinicopathological characteristics, mutation frequencies, and PD-L1 expression. Associations between categorical variables were assessed using the chi-square test or Fisher’s exact test, as appropriate. A p-value of <0.05 was considered statistically significant.

## Results

### Clinicopathological Characteristics

A total of 87 patients diagnosed with NSCLC were enrolled between January 2023 and March 2024. The cohort had a median age of 62 years (range: 26-85), with a male predominance. 64 (73.6%) patients were male, and 23 (26.4%) were female (M: F ratio 2.7:1). Nearly 54% (n=47) were smokers, predominantly male. Adenocarcinoma was evenly distributed among smokers and non-smokers, whereas 77.8% of SCC cases were smokers (p<0.023). Cough was the most common presenting symptom (56.3%), followed by backache/dyspnoea (24.1%), chest pain (19.5%), and haemoptysis/dizziness (11.5%). Histologically, adenocarcinoma was the predominant subtype (n=68, 78.2%), followed by SCC (n=17, 19.5%) and NSCLC-NOS (n=2, 2.3%). Metastases most frequently involved non-regional lymph nodes (79.3%), bones (40.2%), CNS (24.1%), liver (13.8%), and adrenals (11.5%) (Table 2).

**Table 2:** Clinicopathological Characteristics

**Demographics**	
Median age	62 years (range: 26–85)
Gender (M: F)	2.7:1 (64:23)
Tobacco exposure	Smokers: 54% (n=47); Non-smokers: 46% (n=40)
**Clinical Presentation**	
Cough	56.3%
Backache/dyspnoea	24.1%
Chest pain	19.5%
Haemoptysis/dizziness	11.5%
**Histopathology**	
Adenocarcinoma	78.2% (n=68)
Squamous cell carcinoma	19.5% (n=17)
NSCLC-NOS	2.3% (n=2)
**Metastatic Sites**	
Non-regional lymph nodes	79.3%
Bones	40.2%
CNS	24.1%
Liver	13.8%
Adrenals	11.5%

### Molecular Profile

**Figure 1:**
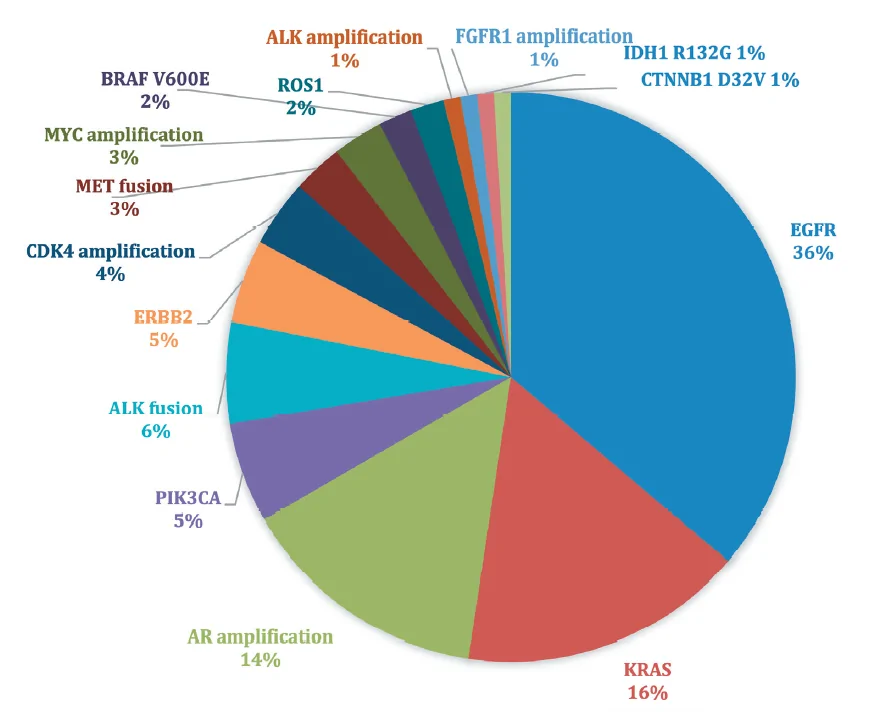
Spectrum of 105 mutations in 87 NSCLC cases

NGS analysis identified a total of 105 molecular alterations across 87 NSCLC cases (Figure 1), with at least one mutation detected in 81.6% (n=71) of patients. All mutation frequencies below are expressed as a proportion of the 105 total alterations unless otherwise specified as per-case rates. The number of mutations per case ranged from one to four: 44 (50.5%) harboured a single mutation, 22 (25.2%) had two mutations, 3 (3.4%) had three, and 2 (2.3%) had four, while 16 (18.4%) showed no detectable molecular alterations. EGFR was the most frequently altered gene (36.2%, n=38), followed by KRAS (16.2%, n=17), AR amplification (14.3%, n=15), PIK3CA (5.7%, n=6), ALK (5.7%, n=6 fusions) and ALK amplification (1.0%,n=1), ERBB2 (4.8%, n=5), CDK4 amplification (3.8%, n=4), MET fusion (2.9%, n=3), MYC amplification (2.9%, n=3), BRAF V600E (1.9%, n=2), ROS1 (1.9%, n=2), FGFR1 amplification (1.0%, n=1), IDH1 R132G (1.0%, n=1), and CTNNB1 D32V (1.0%, n=1). No alterations were detected in HRAS, NRAS, or NTRK1/2. Actionable mutations were detected in 59.8% of cases, defined as molecular alterations with established therapeutic implications, including approved targeted therapies or eligibility for clinical trials encompassing EGFR exon 19-21, ALK fusions, KRAS G12C, ERBB2 exon 20, MET exon 14 skipping, ROS1, and BRAF V600E in our study (Table 3a).

**Table 3a:** Actionable Molecular Alterations

**Actionable Alteration**	**Percentage of Cases (n)**
EGFR exon 19-21	25.70% (27)
ALK fusions	5.71% (6)
ERBB2 exon 20	4.76% (5)
KRAS G12C	3.45% (3)
MET exon 14 skipping	2.85% (3)
ROS1 fusions	1.9% (2)
BRAF V600E	1.9% (2)
RET	0
NTRK	0
**Total Actionable**	**59.82%**

### EGFR Mutations

EGFR mutations were detected in 33 of 87 cases, yielding 38 individual alterations (36.2% of all mutations) (Table 3b). The predominant variants were exon 19 deletions (n=19, 18.1%) and exon 21 L858R substitutions (n=8, 7.6%), followed by EGFR CNV (n=7, 6.6%) and intragenic EGFR::EGFR.E1E8 fusions (n=4, 3.8%). Five patients harboured compound EGFR variants, carrying two distinct EGFR alterations within the same tumour: EGFR CNV co-occurred with either exon 19 deletions or exon 21 L858R in three cases, suggesting amplification of the mutant allele, while one case showed concurrent exon 19 and exon 21 mutations, and another demonstrated an exon 19 deletion with an EGFR::EGFR.E1E8 fusion. All compound EGFR variants were observed exclusively in adenocarcinoma (Table 3c). There was a statistically significant association between EGFR mutations and adenocarcinoma histology: 31 (93.9%) EGFR-mutant cases were adenocarcinomas, whereas only 2 (6.1%) were SCC (p<0.005). EGFR-mutated patients presented at a younger mean age (57.2 years) compared to EGFR wild-type cases (62.1 years).

**Table 3b:** EGFR Mutation Types (38 Mutations in 33 Positive Cases)

**EGFR Mutation Type**	**Number of Mutations**
EGFR exon 19 indel	19
EGFR exon 21 L858R	8
EGFR CNV	7
EGFR fusion (EGFR::EGFR.E1E8)	4
**Total**	**38**

**Table 3c:** Compound EGFR Variants (Multiple EGFR Alterations in a Single Patient)

**Case**	**EGFR Alteration 1**	**EGFR Alteration 2**	**Subtype**
1	EGFR exon 19 SNV	EGFR exon 21 L858R	Adenocarcinoma
2	EGFR exon 21 L858R	EGFR CNV	Adenocarcinoma
3	EGFR exon 19 SNV	EGFR CNV	Adenocarcinoma
4	EGFR exon 21 L858R	EGFR CNV	Adenocarcinoma
5	EGFR exon 19 indel	EGFR::EGFR.E1E8 fusion	Adenocarcinoma

### KRAS Mutations

KRAS was the second most frequent alteration (n=17, 16.2%), with detailed subtypes shown in Table 3d. Over 64.7% of KRAS mutations occurred at codon 12, with G12D the most common variant (n=5), followed by G12C (n=3) and G12R (n=2). Three cases demonstrated KRAS CNV

**Table 3d:** KRAS Mutation Subtypes (n=17)

**KRAS Mutation**	**Number of Cases**
KRAS exon 2 G12D	5
KRAS exon 2 G12C	3
KRAS exon 2 G12R	2
KRAS exon 3 Q61H	2
KRAS exon 2 G12V	1
KRAS exon 2 G13D	1
KRAS CNV	3

### Other Molecular Alterations

AR gene amplification (CNV at ChrX:66766186) was observed in 14.3% (n=15) of cases. Notably, 14 of 15 AR-positive cases presented as co-occurring mutations, co-existing with alterations in EGFR, KRAS, ERBB2, CDK4, CTNNB1, or BRAF. ALK rearrangements were detected in 5.7% of cases, comprising 6 fusions (5 EML4::ALK and 1 FGFR1::ALK) and 1 ALK amplification. PIK3CA mutations were identified in 6 cases (5.7%), including CNV, E545Q, G545K, and E542K (exon 10) and H1047R and H1047Q (exon 21). 2 cases harboured ROS1 fusions (CD74::ROS1 and EZR::ROS1). 5 cases showed ERBB2 mutations (4 with exon 20 p.Gly778_Pro780dup indel and 1 CNV). 3 cases of MET exon 14 skipping were identified.

### Co-Occurring Mutations

Among the 87 cases, 27 (31.0%) harboured two or more concurrent mutations. AR amplification was the most frequent co-occurring alteration, present alongside EGFR in 7 cases and KRAS in 5 cases. CDK4 amplification co-occurred with EGFR in 4 cases. Other notable combinations included EGFR with MYC amplification (n=2), KRAS with PIK3CA (n=2), and a single case each of IDH1 R132G with KRAS G12V, KRAS with MET fusion, ERBB2 with MYC amplification, ALK fusion with FGFR1, and AR with CTNNB1. Four cases demonstrated triple mutations (EGFR + AR + CDK4 in two cases; EGFR + AR + CTNNB1; EGFR + KRAS + CDK4), and one SCC case harboured a quadruple mutation (EGFR + KRAS + PIK3CA + FGFR amplification).

### Distribution of Mutations by Histopathological Subtype

EGFR, ALK, ERBB2, ROS1, BRAF V600E, and MET mutations were found exclusively or predominantly in adenocarcinoma. KRAS was detected in both subtypes, with a proportionally higher rate in SCC (23.5%) than adenocarcinoma (17.6%). No actionable mutations other than KRAS were observed in SCC (Table 4).

**Table 4:** Distribution of Molecular Mutations by Histopathological Subtype

**Molecular Mutation**	**Total cases (n)**	**Adenocarcinoma (n=68)**	**SCC (n=17)**	**NSCLC-NOS (n=2)**
EGFR	33	31 (45.6%)	2 (11.8%)	0 (0%)
KRAS	17	12 (17.6%)	4 (23.5%)	1 (50.0%)
ALK fusion	6	6 (8.8%)	0 (0%)	0 (0%)
ERBB2 exon 20	5	5 (7.4%)	0 (0%)	0 (0%)
ROS1	2	2 (2.9%)	0 (0%)	0 (0%)
BRAF V600E	2	2 (2.9%)	0 (0%)	0 (0%)
MET exon 14 skipping	3	3 (4.4%)	0 (0%)	0 (0%)
RET	0	0 (0%)	0 (0%)	0 (0%)
NTRK	0	0 (0%)	0 (0%)	0 (0%)

### PD-L1 Expression

PD-L1 expression was evaluated in 70 of 87 cases; data were unavailable for 17 patients (19.5%). Among the 70 evaluable cases, PD-L1 positivity (TPS ≥1%) was detected in 45.7% (n=32), while 54.3% (n=38) were negative (TPS <1%). Of the 32 PD-L1–positive cases, 19 (59.4%) demonstrated weak expression (TPS 1–49%) and 13 (40.6%) showed strong expression (TPS ≥50%) (Table 5a).

**Table 5a:** PD-L1 Expression and Association with Molecular Subtypes

**PD-L1 Status**	**Proportion**
PD-L1 Negative (<1%)	54.3% (38/70)
PD-L1 Positive (≥1%)	45.7% (32/70)
Weak positive (1–49%)	59.4% of positive (19/32)
Strong positive (≥50%)	40.6% of positive (13/32)
**By Molecular Subtype**	**PD-L1 Positivity**
EGFR wild-type	51.0% (25/49)
EGFR mutant	23.7% (9/38)
KRAS mutant	57.1%
ALK mutant	66.7% (4/6)

When stratified by histopathological subtype, PD-L1 positivity was higher in SCC (50.0%, 6/12 evaluable) compared to adenocarcinoma (44.6%, 25/56 evaluable). Strong PD-L1 expression (≥50%) was observed in 17.9% of adenocarcinomas and 16.7% of SCCs, while weak expression (1- 49%) was more frequent in SCC (33.3%) than in adenocarcinoma (26.8%) (Table 5b).

**Table 5b:** PD-L1 Expression by Histopathological Subtypes

**PD-L1 Status (TPS)**	Adenocarcinoma (n=56*)	SCC (n=12*)	NSCLC-NOS (n=2*)
Negative (<1%)	31 (55.4%)	6 (50.0%)	1 (50.0%)
Weak Positive (1–49%)	15 (26.8%)	4 (33.3%)	0 (0%)
Strong Positive (≥50%)	10 (17.9%)	2 (16.7%)	1 (50.0%)
**Total Positive (≥1%)**	**25 (44.6%)**	**6 (50.0%)**	**1 (50.0%)**
Not Available	12	5	0
**Total Cases**	**68**	**17**	**2**

Distinct patterns of PD-L1 expression were observed across molecular subtypes. PD-L1 positivity was markedly lower in EGFR-mutant cases (23.7%, 9/38) compared to EGFR wild-type cases (51.0%, 25/49), suggesting reduced immunogenicity in EGFR-driven tumours. This inverse relationship was consistent within adenocarcinoma, where only 19.2% (5/26) of EGFR-mutant cases were PD-L1 positive versus 48.3% (14/29) of EGFR wild-type cases. Conversely, KRAS-mutant tumours demonstrated higher PD-L1 positivity (57.1%), with 35.7% exhibiting strong expression; KRAS-mutant adenocarcinomas showed particularly high PD-L1 positivity (66.7%, 6/9) compared to KRAS wild-type adenocarcinomas (28.3%, 13/46). ALK-positive tumours demonstrated PD-L1 positivity in 66.7% (4/6) of cases, with 50.0% (2/4) of evaluable ALK-mutant adenocarcinomas expressing PD-L1. Additionally, 62.5% of tumours harbouring no actionable mutations expressed PD-L1 (Table 8, Table 9, Table 5c).

**Table 5c:** PD-L1 Expression by Mutation Status and Histopathological Subtype

**Mutation Status**	**Subtype**	**Evaluable (n)**	**PD-L1 Positive**	**PD-L1 Negative**
EGFR Mutant	Adenocarcinoma	26	5 (19.2%)	21 (80.8%)
EGFR Mutant	SCC	1	1 (100%)	0 (0%)
EGFR Wild-type	Adenocarcinoma	29	14 (48.3%)	15 (51.7%)
EGFR Wild-type	SCC	11	5 (45.5%)	6 (54.5%)
KRAS Mutant	Adenocarcinoma	9	6 (66.7%)	3 (33.3%)
KRAS Mutant	SCC	2	1 (50.0%)	1 (50.0%)
KRAS Wild-type	Adenocarcinoma	46	13 (28.3%)	33 (71.7%)
KRAS Wild-type	SCC	10	5 (50.0%)	5 (50.0%)
ALK Mutant	Adenocarcinoma	4	2 (50.0%)	2 (50.0%)
ALK Mutant	SCC	0	-	-
ALK Wild-type	Adenocarcinoma	51	17 (33.3%)	34 (66.7%)
ALK Wild-type	SCC	12	6 (50.0%)	6 (50.0%)

*Evaluable cases exclude those with PD-L1 data not available. - indicates no evaluable cases.*

## Discussion

Comprehensive molecular profiling is critical for guiding personalised therapy in advanced NSCLC, and NGS enables simultaneous detection of multiple actionable mutations from limited tissue. In this single-centre retrospective study, 87 NSCLC patients underwent NGS using the Oncomine Focus Assay (52-gene panel) alongside PD-L1 assessment between January 2023 and March 2024.

The demographic profile of our cohort, with a median age of 62 years and male predominance (M: F 2.7:1), is consistent with prior Indian data from Gupta et al. (median 61.3 years, M: F 1.9:1) and the South Indian study by Jacob et al. (median 60.3 years) (16,17). Cough was the most common presenting symptom, followed by dyspnoea and chest pain, aligning with the large North Indian series reported by Mohan et al. (18). The metastatic distribution, with non-regional lymph nodes (79.3%) and bones (40.2%) as the most common sites, was also concordant with previous reports (17,18). Histopathologically, adenocarcinoma predominated (78.2%), followed by SCC (19.5%), which mirrors both Indian and Western literature (10,17,19).

### Mutational Landscape

Overall, 81.6% of patients harboured at least one mutation, and actionable alterations were identified in 59.8%. EGFR was the most frequent driver (36.2%), a finding concordant with other Indian studies by Aggarwal et al. (38.4%) and Gupta et al. (32.4%) (16,20), and higher than Western cohorts where KRAS predominates (19). The predominance of exon 19 deletions (18.1%) followed by exon 21 L858R (7.6%) aligns with the well-established pattern reported by Sharma et al. and Sakata et al. (10,21). Notably, EGFR CNVs and intragenic EGFR::EGFR.E1E8 fusions accounted for 10.5% of all EGFR alterations, and CNVs frequently co-occurred with exon 19 or L858R mutations as compound variants, suggesting potential clinical implications for treatment response that merit further investigation.

KRAS was the second most prevalent alteration (16.2%), comparable to Asian data from Gupta et al. (18.2%) and Rajadurai et al., but lower than the 24.5% reported in the Western cohort by Forsythe et al. (16,19,22). Consistent with earlier reports, over 64.7% of KRAS mutations occurred at codon 12, with G12C the most common variant (23). This is clinically significant given the recent approval of sotorasib for KRAS G12C-mutant NSCLC.

AR gene amplification was detected in 14.3% of cases, higher than the 1.77% reported by Xie et al. (23). Notably, 14 of 15 AR-mutant cases co-existed with other driver alterations (EGFR, KRAS, ERBB2, CDK4, CTNNB1, or BRAF), consistent with findings by Wang et al., who demonstrated KRAS-dependent crosstalk with AR signalling in NSCLC cell lines (24).

PIK3CA mutations (5.7%) were consistent with Asian and Western reports from Rajadurai et al. and Simarro et al. (22,25), though higher than the 1.2% reported by Gupta et al. (16). ALK rearrangements (6.7%), predominantly EML4::ALK fusions, were comparable to the 6% reported by Rajadurai et al. (22), with a single novel ALK::FGFR1 fusion observed. ERBB2 alterations (4.8%), MET exon 14 skipping (2.9%), and BRAF V600E (1.9%) were all consistent with published frequencies from Indian studies (16,20). ROS1 fusions were observed in 1.9% of cases, with CD74 and EZR as fusion partners; both patients were female non-smokers with adenocarcinoma, aligning with the established epidemiological profile of ROS1-rearranged NSCLC (26). A single IDH1 R132G mutation co-occurred with KRAS G12V in a 65-year-old male, consistent with literature associating IDH active-site mutations with male sex, older age, and coexisting KRAS mutations (27). No alterations were observed in HRAS, NRAS, or NTRK1/2 (Table 6).

### Co-Occurring and Compound Mutations

A notable finding of this study is the co-occurring mutations, with 31.0% of cases harbouring two or more concurrent molecular alterations. The pattern was the co-occurrence of AR amplification with various driver mutations in 14 of 15 AR-positive cases, particularly with EGFR exon 19 deletions (n=5) and KRAS exon 2 variants (n=3). Compound EGFR variants (CNV co-occurring with exon 19 or L858R) were also observed, potentially reflecting clonal heterogeneity or gene amplification of the mutant allele. The co-occurrence of IDH1 R132G with KRAS G12V is consistent with the known association between these two alterations (27). These findings highlight the clinical importance of comprehensive multi-gene NGS panels that can detect co-occurring variants, which may influence treatment selection and resistance patterns.

### Distribution of Mutations by Histopathological Subtype

EGFR mutations were significantly more frequent in adenocarcinomas (93.9%) than in SCC (6.1%, p<0.005), consistent with the well-established association between EGFR alterations and adenocarcinoma histology (1,2).

### PD-L1 Expression and its Association with Molecular and Histopathologic Subtypes

PD-L1 expression was detected in 45.7% of evaluable cases, comparable to the 44.7% reported by Kilaru et al. from another Indian centre (28). When stratified by histopathological subtype, PD-L1 positivity was higher in SCC (50.0%) compared to adenocarcinoma (44.6%), a finding consistent with several studies reporting higher PD-L1 expression in squamous histology (28,29). This may be attributed to the higher tumour mutational burden and smoking-related immunogenicity typically associated with SCC. However, strong PD-L1 expression (≥50%) was similar across both subtypes (17.9% in adenocarcinoma vs. 16.7% in SCC), suggesting that while overall positivity differs, the proportion of high expressors remains comparable (30).

The inverse relationship between EGFR mutations and PD-L1 expression observed in our cohort (23.7% in EGFR-mutant vs. 51.0% in EGFR wild-type) is consistent with findings from Onur et al. and a meta-analysis by Lan et al., supporting the concept that EGFR-driven cancers exhibit reduced immunogenicity (29,31). This pattern was particularly evident within adenocarcinomas, where only 19.2% of EGFR-mutant cases were PD-L1 positive compared to 48.3% of EGFR wild-type cases. This has direct therapeutic implications, as EGFR-mutant patients are less likely to benefit from immune checkpoint inhibitors as first-line therapy and should be prioritised for targeted TKI treatment.

Conversely, KRAS-mutant tumours showed higher PD-L1 expression (52.9%) compared to KRAS wild-type cases (29.4%), a pattern consistent with the Danish cohort study by Cronin-Fenton et al. (32). This association is biologically plausible, as KRAS mutations have been shown to upregulate PD-L1 expression through p-ERK signalling, promoting an inflammatory tumour microenvironment that may explain the clinical benefit of immune checkpoint inhibitors observed in KRAS-mutant NSCLC (33). Notably, KRAS-mutant adenocarcinomas demonstrated the highest PD-L1 positivity (66.7%) among all mutation–subtype combinations, compared to just 28.3% in KRAS wild-type adenocarcinomas, further supporting the potential role of immunotherapy in this molecular subgroup. ALK-rearranged tumours demonstrated PD-L1 positivity in 66.7% of cases (4/6), consistent with findings by Kim et al. and D’Incecco et al. (34,35), though the small sample size limits definitive conclusions. Notably, 62.5% of tumours without actionable mutations also expressed PD-L1, reinforcing the role of immunotherapy as a treatment option for patients lacking targetable molecular alterations.

**Table 6:** Comparison of NGS data in lung adenocarcinoma studies from global studies

**Article reference**	**TCGA Portal Data Base **(36)	**D’Haene N et al **(37)	**DiBardino et al **(38)	**Gupta et al **(16)	**Forsythe** **et al **(19)	**Simarro** **et al **(25)	**Rajadurai** **et al **(22)		**Aggarwal** **et al **(20)	**Present study**
Year of study	2014	2015	2017	2018	2020	2023	2023		2023	2024
Total sample	230	223	22	154	799	350	NEXUS 1	NEXUS 2-3	552	87
							469			
Total n.o of mutations	NA	137	204	180	NA	191	293		320	105
Range of mutation	NA	NA	4-24	1-3	NA	2-4	NA		NA	1-4
Most frequent mutation	KRAS	KRAS	EGFR	EGFR	KRAS	KRAS	EGFR	EGFR	EGFR	EGFR
EGFR	11%	11.60%	27.27%	32.40%	6.96%	15.3%	45%	47%	38.41%	36.19%
KRAS	32%	35.90%	18.10%	18.18%	24.49%	26.60%	17%	13%	NA	16.19%
AR	NA	NA	NA	NA	NA	NA	NA	0.30%	NA	14.28%
ALK	NA	NA	NA	NA	0.12%	4%	1.90%	6%	12.14%	6.66%
PIK3CA	7%	3.60%	4.50%	1.20%	1.20%	5.40%	8%	7%	NA	5.71%
MYC	NA	NA	NA	NA	NA	2%	NA	NA	NA	2.85%
ERBB2	1.70%	0.90%	NA	3.20%	0	NA	0.60%	8%	NA	4.59%
CDK4	4%	NA	16.30%	5.10%	NA	NA	NA	2.20%	NA	4.59%
MET	4	NA	NA	0	NA	3.60%	1.90%	9%	1.09%	2.85%
ROS	NA	NA	NA	NA	NA	1.40%	NA	2.20%	3.62%	2.29%
BRAF	7%	6.30%	NA	1.90%	1.08%	6.90%	1.90%	1%	2.72%	1.90%
FGFR	NA	NA	NA	NA	NA	NA	1.30%	4%	NA	1.90%
CTNNB1	NA	NA	NA	3.20%	NA	NA	6%	1.3%	NA	0.95%

## Conclusion

This single-centre retrospective study highlights the importance of integrating NGS-based molecular testing with PD-L1 expression analysis in the comprehensive molecular profiling of NSCLC. NGS identified 105 alterations across 87 cases, predominantly EGFR (36.2%), KRAS (16.2%), and AR amplification (14.3%). Actionable mutations were detected in 59.8%, led by EGFR exon 19-21 (25.7%), ALK fusions (5.7%), ERBB2 exon 20 (4.8%), KRAS G12C (3.5%), MET exon 14 skipping (2.9%), BRAF V600E and ROS1 (1.9% each). Co-occurring mutations were observed in 31.0% of patients, with AR amplification frequently co-existing with other driver alterations, underscoring the molecular complexity of NSCLC.

PD-L1 expression was detected in 45.7% of cases, with distinct patterns across molecular subtypes. PD-L1 positivity was lower in EGFR-mutant tumours compared to EGFR wild-type, supporting the reduced immunogenicity of EGFR-driven cancers. Conversely, KRAS-mutated and ALK-rearranged tumours demonstrated higher PD-L1 expression, suggesting potential sensitivity to immune checkpoint inhibition.

These findings reinforce the need for combined molecular and immunohistochemical profiling to optimise personalised treatment strategies in NSCLC. The coexistence of actionable mutations and variable PD-L1 expression highlights the evolving role of tailored therapeutic sequencing, integrating targeted therapies and immunotherapies based on individual molecular and immunological profiles.

### Limitations

This study has several limitations that should be acknowledged. First, the relatively small sample size (n=87) from a single centre limits the generalisability of the findings and may reduce the statistical power to detect associations in low-frequency mutations. Second, PD-L1 expression data were unavailable for 17 cases (19.5%), introducing potential selection bias. Third, the study population was drawn from a single tertiary care centre, which may not fully represent the broader Indian NSCLC population. Larger, multi-centre studies with survival analysis are warranted to validate these observations and their clinical implications.

## Ethical Approval

The study was approved by the Institutional Ethics Committee, which is organised and operates in accordance with ICMR guidelines, ICH-GCP standards, and the New Drugs and Clinical Trial Rules (2019). Ethics Committee Reference No.: 1467/2022.

## Conflict of Interest

The authors report there are no competing interests to declare.
